# Comparison of Surgical Outcomes Between Single-Port Access Laparoscopic and Single-Site Robotic Surgery in Benign Gynecologic Diseases: A Single-Center Cohort Study

**DOI:** 10.3390/jcm14030799

**Published:** 2025-01-26

**Authors:** Suk Hwan Hyun, Ji Geun Yoo, Ye Won Jung, Won Kyo Shin, Soo Youn Song, Jae Sung Choi, Young Bok Ko, Mina Lee, Byung Hun Kang, Mia Park, You Jin Kim, Geon Woo Lee, Kyong-No Lee, Heon Jong Yoo

**Affiliations:** 1 Department of Obstetrics & Gynecology, Chungnam National University School of Medicine, 266, Munhwa-ro, Jung-gu, Daejeon 35015, Republic of Korea; neo1714@naver.com (S.H.H.); wonyberry@naver.com (Y.W.J.); bluered120@gmail.com (W.K.S.); sysong@cnuh.co.kr (S.Y.S.); cjs570@hanmail.net (J.S.C.); koyoung27@gmail.com (Y.B.K.); minari73@cnuh.co.kr (M.L.); missinglime@cnuh.co.kr (B.H.K.); mia86@cnuh.co.kr (M.P.); niguemai@cnuh.co.kr (Y.J.K.); gunu1981@naver.com (G.W.L.); kyongnolee@cnuh.co.kr (K.-N.L.); 2 Department of Obstetrics and Gynecology, Daejeon St. Mary’s Hospital, College of Medicine, The Catholic University of Korea, Seoul 34943, Republic of Korea; jgyoo@catholic.ac.kr

**Keywords:** adnexal surgery, hysterectomy, laparoscopic surgery, myomectomy, robotic surgery, single-port

## Abstract

**Background/Objectives:** To compare the outcomes of single-port access laparoscopic surgery (SPALS) and single-site robotic surgery (SSRS) for benign gynecological diseases, we retrospectively analyzed clinical data from 367 patients who underwent gynecologic surgery at Sejong Chungnam National University Hospital from October 2020 to December 2023. **Methods**: Of these 367 patients, 197 underwent SPALS, whereas 170 underwent SSRS. The SPALS group comprised 87 patients who underwent hysterectomy; 107, adnexal surgery (cystectomy: 44, adnexectomy: 63); and 1 myomectomy. The SSRS group included 68 patients who underwent myomectomy; 61, adnexal surgery (cystectomy: 52, adnexectomy: 9); 35, hysterectomy, and 4, sacrocolpopexy. **Results**: Both surgical techniques were successful, with no patients requiring open surgery. No significant differences were observed in the baseline characteristics between the two groups. Compared with the SPALS group, the SSRS group had a younger age (39.8 ± 9.5 vs. 44.5 ± 12.3 years, *p* = 0.001). Severe intra-abdominal adhesions were more frequently observed in the SSRS group (*p* = 0.004). Operation time (118.1 ± 65.9 vs. 57.1 ± 27.3 min, *p* = 0.001), gas passing time (39.4 ± 15.37 vs. 30.4 ± 13.5 h, *p* = 0.001), and hospital length of stay (4.26 ± 1.02 vs. 4.02 ± 0.8 days, *p* = 0.012) were significantly longer in the SSRS group. However, no significant differences were found between the two groups in terms of intraoperative blood loss, intraoperative complications, or readmission rates. **Conclusions**: SSRS offers a feasible and promising approach for treating gynecological benign diseases. Younger and lower-parity patients tend to undergo SSRS rather than SPALS, with SSRS primarily performed for myomectomy and complex adnexal surgery. However, operation time and gas passing time were significantly longer in the SSRS group.

## 1. Introduction

Minimally invasive surgery has emerged as a significant alternative to conventional laparotomy in gynecological procedures. The different minimally invasive approaches include laparoscopic, robotic, and natural orifice transluminal surgeries [1]. Among these, conventional laparoscopic surgery, which employs 3 to 4 ports, has been widely practiced. However, the recent introduction of single-site surgery has shifted the focus due to its notable advantages, including reduced postoperative pain, a shorter hospital stay, rapid recovery, and smaller scars [2,3]. Despite its cosmetic benefits, single-site laparoscopic surgery presents challenges, including limited triangulation, reduced visualization depth, and higher likelihood of instrument clashes and crowding compared with conventional multiport laparoscopy [4]. Additionally, the steep learning curve poses a significant barrier to surgeons aiming to master this technique [5]. Recently, robotic single-site surgery has gained traction and popularity in gynecologic surgery [6]. This approach offers several advantages, such as three-dimensional (3D) visualization, wristed instruments with an enhanced range of motion, improved ergonomics, enhanced surgeon comfort, and the ability to perform more complex procedures [7]. It is considered an alternative to address the limitations of single-site laparoscopic surgery. However, despite these potential benefits, evidence directly comparing the clinical outcomes of single-site laparoscopic surgery and single-site robotic surgery in benign gynecologic conditions remains limited. Therefore, this study aimed to compare the outcomes of single-site laparoscopic surgery and single-site robotic surgery for benign gynecologic diseases and to determine whether single-site robotic surgery can serve as a viable alternative.

## 2. Materials and Methods

**Figure 1:**
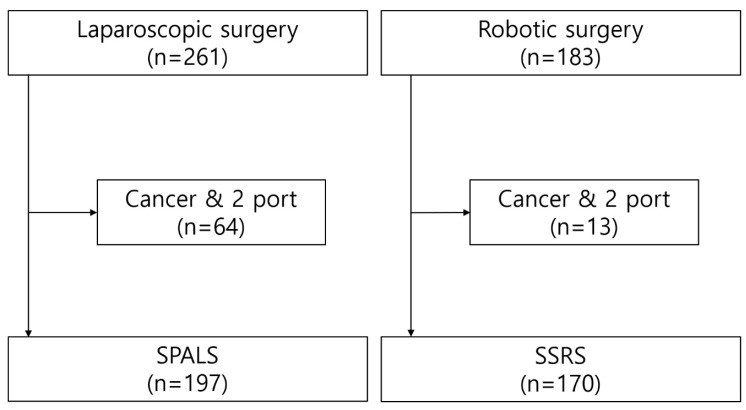
Flowchart of the participant selection process.

This retrospective study was conducted at the Sejong Chungnam National University Hospital and was approved by the hospital’s Institutional Review Board (2024-10-005). Between July 2020 and December 2023, 261 laparoscopic surgeries and 183 robotic surgeries were performed. Figure 1 shows the participant selection process. After excluding patients who were diagnosed with cancer and underwent surgical procedures that used more than one port, 197 patients were included in the single-port access laparoscopic surgery (SPALS) group and 170 in the single-site robotic surgery (SSRS) group.

Table 1 shows the indications for surgery. The SPALS group comprised 87 patients who underwent hysterectomy; 50, adnexectomy; 30, ovarian cystectomy; 13, salpingectomy; 6, hemorrhagic cyst coagulation; 2, cornual resection; 1, myomectomy; and 8, other surgical procedures. The SSRS group comprised 67 patients who underwent myomectomy; 50, ovarian cystectomy; 34, hysterectomy; 9, adnexectomy; 4, sacrocolpopexy; 1 tuboplasty; and 2, other surgical procedures.

Data on the patients’ clinical characteristics and perioperative outcomes were retrospectively collected by reviewing the patients’ medical records. The clinical characteristics included age, body mass index (BMI), parity, history of previous abdominal surgery, indication for surgery, and the presence of pelvic adhesions. The perioperative outcomes included total operation time, changes in hemoglobin (Hb) levels after surgery, postoperative pain score, surgery-to-gas passing time (hours), intraoperative complications, wound complication rate, and hospital stay duration. Pelvic adhesions were categorized according to their severity as none, mild, moderate, or severe. Operative time was defined as the period from the start of the skin incision to the completion of skin closure. A change in Hb levels after surgery was defined as the difference between the preoperative Hb level and the Hb level measured on the first postoperative day. The level of postoperative pain was evaluated using the numeric rating scale. The different intraoperative complications included injuries to adjacent organs, such as the bladder, bowel, vessels, and nerves. Wound complications included wound dehiscence, evisceration, and hernia. Hospital stay was defined as the duration from the day of surgery to the day of discharge. In South Korea, the National Health Insurance (NHI) system partially covers the total medical expenses, while the remaining costs are borne by the patient. The term “medical cost” specifically refers to the out-of-pocket expenses paid by the patient at discharge, rather than the total medical expenses. The different fertility-preserving surgical procedures included myomectomy, ovarian cystectomy, paratubal cystectomy, adenomyomectomy, and tuboplasty.

The advantages and disadvantages of both surgical methods were thoroughly explained to the patients, who then selected the surgical method and provided informed consent. This decision was made without the influence of the surgeon’s preference.

### 2.1. Surgical Procedure

SPALS and SSRS were performed in the lithotomy position by three well-trained gynecologic surgeons. A single 1.5 to 2 cm vertical incision was made at the outer limit of the umbilical folds. The base of the umbilicus was exposed through blunt dissection, and a scalpel was used to create a fascial incision while lifting the umbilicus. The commercial port for single-port surgery was inserted only after confirming the absence of attachments or adhesions. A pneumoperitoneum was established with CO_2_, with the pressure set at up to 14 mmHg. The patient was placed in the reverse Trendelenburg position with a slight left lateral decubitus during the surgical procedure. Subsequently, the SPALS was performed using laparoscopic instruments, whereas SSRS was carried out after docking the da Vinci^®^ Xi surgical system (Intuitive Surgical, Sunnyvale, CA, USA). Overall, the surgical procedures performed by the three surgeons did not differ.

### 2.2. Statistical Analysis

The distributions of the patient characteristics between the SPALS and SSRS groups were compared using the *t*-test for continuous variables (or the Wilcoxon rank sum test when the expected frequency within any cell was less than 5) and the χ^2^ test (or Fisher’s exact test when the expected frequency within any cell was less than 5) for categorical variables. Statistical analyses were performed using Statistical Package for the Social Sciences software (version 12.0; SPSS, Chicago, IL, USA). A two-sided *p* value of <0.05 was considered significant.

## 3. Results

The patients’ baseline characteristics are presented in Table 2. No significant difference was found between the two groups in terms of diabetes incidence (1.5% vs. 3.5%, *p* = 0.313), BMI (23.4 ± 3.64 vs. 22.9 ± 3.76 kg/m^2^, *p* = 0.191), and previous abdominal surgery history (44.7% vs. 37.1%, *p* = 0.167). Compared with the SPALS group, the SSRS group had a younger age (44.5 ± 12.3 vs. 39.8 ± 9.54 years, *p* = 0.001) and lower parity (1.49 ± 1.09 vs. 1.11 ± 1.03, *p* = 0.08). Additionally, the SSRS group had a higher incidence of severe intra-abdominal adhesion (9.5% vs. 15.0%, *p* = 0.004).

In the present study, none of the patients underwent open surgery. The perioperative outcomes are shown in Table 3. No significant difference was found between the two groups in terms of Hb change after surgery (1.36 ± 1.05 vs. 1.5 ± 1.00, *p* = 0.211), postoperative pain score (2.8 ± 1.09 vs. 2.92 ± 1.10, *p* = 0.114), intraoperative complications (2% vs. 1.8%, *p* = 0.853), wound dehiscence (2 vs. 5, *p* = 0.336), wound hernia (1 vs. 2, *p* = 0.898), and readmission rates within 1 month (1.52% vs. 0%, *p* = 0.301). Operation time (57.1 ± 27.28 vs. 118.1 ± 65.95 min, *p* = 0.001), surgery-to-gas passing time (30.4 ± 13.53 vs. 39.4 ± 15.367 h, *p* = 0.001), and length of hospital stay (4.02 ± 0.82 vs. 4.26 ± 1.02 days, *p* = 0.012) were significantly longer in the SSRS group. The medical cost for SPALS was $1170 ± 492, whereas the cost for SSRS was $7221 ± 684 (*p* < 0.001). Additionally, the number and frequency of fertility-preserving surgical procedures were significantly higher in the SPALS group (n = 37, 18.78%) compared with the SSRS group (n = 120, 70.58%) (*p* < 0.001).

As shown in Table 4, the univariate analysis indicated that parity had a significant effect on total operation time (regression coefficient: −5.791, *p* = 0.038), whereas diabetes and adhesion significantly affected the surgery-to-gas-passing time (regression coefficients: 15.779 and 2.126, *p* = 0.004 and *p* = 0.007, respectively). The other variables did not show significant effects on either outcome. A multivariate analysis was subsequently performed to adjust for these significant variables. After adjustment, the operation type remained a significant factor influencing the total operation time (*p* = 0.001), whereas diabetes, intra-abdominal adhesions, and operation type were affected by gas-passing time (*p* = 0.019, *p* = 0.050, and *p* = 0.001, respectively).

## 4. Discussion

In the present study, younger patients, those with low parity, and those with suspected severe intra-abdominal adhesions tended to undergo SSRS. In South Korea, SPALS is covered by NHI, making it a more cost-effective option ($1170 ± 492), whereas SSRS is not covered by NHI, making it a more expensive option ($7221 ± 684). Despite the higher cost of SSRS than that of SPALS, patients in need of fertility-preserving surgery such as myomectomy, ovarian cystectomy, paratubal cystectomy, adenomyomectomy, and tuboplasty showed a preference for SSRS over SPALS. This preference may be attributed to the perception that robotic surgery offers improved precision and outcomes [8]. In this study, no significant differences were observed in most operative outcomes, except for operation time, hospital stay, and gas passing time, between SPALS and SSRS for benign gynecologic diseases. In the present study, SSRS was feasible for treating benign gynecologic diseases. This method may be more desirable for patients who want to undergo fertility-preserving surgery.

Longer operation times and surgery-to-gas passing times were observed in the SSRS group compared with the SPALS group in this study. Robotic surgery involves additional procedures such as docking time, instrument changes, and camera cleaning, which could contribute to the overall longer duration of the operation. Additionally, the higher proportion of more complex surgical procedures, such as fertility-preserving surgery, in the SSRS group may have contributed to the longer operation times. Kim et al. also reported that SSRS was associated with longer operation times without significant differences in postoperative bleeding or complications of ovarian cystectomy (surgical time: 96.96 ± 46.23 vs. 130.41 ± 49.59 min, *p* < 0.001; Hb level reduction: 1.65 ± 0.93 vs. 1.60 ± 1.10, *p* = 0.812) [9]. Similarly, Lee et al. found that the mean operation time was longer for SSRS (89.0 ± 26.7 vs. 95.4 ± 33.2 min, *p* = 0.01), although no significant differences were found in post-operative anti-Müllerian hormone levels (2.53 ± 2.07 vs. 2.49 ± 1.58 ng/mL, *p* > 0.05) [10]. Many studies have reported that intra-abdominal adhesions and postoperative hyperglycemia are associated with postoperative bowel recovery. Antosh DD et al. demonstrated that patients who underwent adhesiolysis had a significantly higher incidence of postoperative ileus (odds ratio (OR): 1.7, 95% confidence interval (CI): 1.03–2.83) [11]. Similarly, Li et al. reported that adhesiolysis was a significant independent risk factor for postoperative ileus in patients undergoing hysterectomy for benign indications (OR of 1.818, 95% CI: 1.146–2.885, *p* = 0.011) [12]. In a systematic review and meta-analysis, Hou et al. confirmed that adhesiolysis significantly increases the risk of postoperative ileus in patients undergoing hysterectomy (OR: 1.97, 95% CI: 1.52–2.56) [13]. Separately, Ozdemir et al. reported that diabetes mellitus is an independent risk factor for postoperative ileus in patients undergoing robot-assisted radical prostatectomy (OR: 36.96, 95% CI: 2.10–649.56) [14]. In the present study, diabetes, intra-abdominal adhesions, and operation type affected the postoperative gas-passing time. To the best of our knowledge, some studies have reported no difference in postoperative bowel recovery between the SPALS and SSRS groups [15,16]. For example, Won S et al. reported that, although the SSRS group had a significantly longer operation time compared with the SPALS group (117.5 ± 44.8 vs. 145.8 ± 53.7 min, *p* < 0.001), no significant difference was found in the incidence of postoperative ileus between the two groups (SPALS: 1 patient, SSRS: 0 patient, *p* = 0.309) [15]. Similarly, Shin et al. also reported that, although robotic gastrectomy had a significantly longer operation time compared with laparoscopic gastrectomy (148.32 ± 45.24 vs. 180.47 ± 47.63 min, *p* < 0.001), no statistically significant difference was found in the incidence of postoperative ileus between the two groups (3.9% vs. 2.4%, *p* = 0.340) [16]. No studies have demonstrated a significant difference between the two groups. These results should be further verified in studies with a large prospective cohort.

In the present study, patients in the SSRS group had longer hospital stays compared with those in the SPALS group; however, other studies have demonstrated the feasibility and safety of SSRS for gynecological surgical procedures without increasing the length of hospital stay (2.72 ± 0.98 vs. 2.55 ± 0.99 days, *p* = 0.446) (2 vs. 2 days, *p* = 0.391) [9,17]. Some studies have even reported that SSRS resulted in a shorter hospital stay compared with SPALS and concluded that robotic myomectomy is a feasible and safe option for gynecological diseases (5.4 ± 1.0 vs. 4.7 ± 0.9 days, *p* = 0.001) [15]. By contrast, Seo et al. observed a longer operation time and hospital stay in the SSRS group (operation time 77.5 ± 47.1 vs. 176.1 ± 59.0 min, *p* < 0.0001; hospital stay 3.7 ± 0.8 vs. 4.1 ± 0.7 days, *p* = 0.0100) [18]. The length of hospital stay may be influenced by the variations in health insurance systems across different institutions.

Robotic surgery offers several advantages over conventional laparoscopic surgery in various aspects. First, robotic surgery significantly enhances endoscopic suturing due to its highly articulated, wrist-like instruments and 3D high-definition visualization, allowing for precise manipulation in confined or challenging anatomical spaces. These features minimize technical difficulty and improve the accuracy of suturing compared with conventional laparoscopy. This is one of the key reasons why robotic myomectomy is widely accepted and increasingly popular [19]. In addition to suturing, robotic surgery provides distinct benefits for patients with a high BMI. Laparoscopic surgery in obese patients poses challenges such as limited visualization, difficulty in achieving adequate peritoneal distension, and increased risks associated with Trendelenburg positioning. Robotic surgery addresses these limitations through enhanced 3D visualization, articulated instruments for greater mobility, and stable intra-abdominal pressure, enabling greater precision and reducing the conversion rate to open surgery. These advantages make robotic surgery a valuable option for patients with high BMI, improving surgical outcomes and safety. Eddib A et al. found that, although morbid obesity (BMI > 35) was associated with increased operative time in robotic-assisted gynecologic surgery, no significant differences were observed in blood loss, complications, length of hospital stay, or pain medication usage between morbidly obese and non-obese patients, demonstrating the feasibility and safety of robotic surgery in high BMI populations [20]. Similarly, robotic surgery offers substantial benefits in managing large uteri. The articulated instruments provide enhanced precision and control, facilitating better dissection in confined spaces. Additionally, robotic platforms reduce blood loss and the likelihood of conversion to open surgery, even in cases of large uterine size, providing a safer and more effective minimally invasive option. Nozaki T et al. compared robotic-assisted hysterectomy (RAH) and total laparoscopic hysterectomy for large uteri and reported that RAH was associated with significantly lower blood loss and shorter operative times, particularly in patients with a uterine weight of ≥750 g. These findings demonstrate the safety and efficiency of RAH in managing large uteri [21]. Collectively, they highlight the versatility and effectiveness of robotic surgery in addressing complex surgical challenges, making it a valuable and reliable option for a diverse range of patient populations and clinical scenarios.

Our study has some limitations. First, the retrospective design may have introduced selection bias, potentially affecting the results. The absence of patient matching between the groups may have resulted in imbalances in baseline characteristics, such as age, parity, and pathology severity, making it difficult to attribute outcomes solely to the surgical techniques. Future studies using propensity score matching or randomized trials are needed to reduce these biases and enhance comparability. Second, although the surgical approaches were performed by three experienced surgeons, and although the overall procedures were standardized, variations in individual techniques could have influenced the outcomes. Third, the heterogeneity in the indications for surgery may limit the validity of comparing the mean operation times. Fourth, this study focused on the short-term surgical outcomes. Hence, future studies should include long-term assessments, particularly those related to fertility outcomes.

## 5. Conclusions

SSRS appears to be a viable option for treating benign gynecologic conditions. Younger patients and those with lower parity were more likely to undergo SSRS, which is particularly suited for myomectomy and complex adnexal surgery. Therefore, the operation and gas-passing times were longer for SSRS.

## Figures and Tables

**Table 1:** Indications for surgery.

	SPALS (n = 197)	SSRS (n = 170)
Myomectomy	1 (0.51%)	67 (39.41%)
Ovarian cystectomy	30 (15.23%)	50 (29.41%)
Paratubal cystectomy	0 (0.00%)	2 (1.18%)
Adenomyomectomy	0 (0.00%)	1 (0.59%)
Tuboplasty	0 (0.00%)	1 (0.59%)
Hemorrhagic cyst coagulation	6 (3.05%)	0 (0.00%)
Hysterectomy	87 (44.16%)	34 (20.00%)
Adnexectomy	50 (25.38%)	9 (5.29%)
Salpingectomy	13 (6.60%)	0 (0.00%)
Cornual resection	2 (1.02%)	0 (0.00%)
Sacrocolpopexy	0 (0.00%)	4 (2.35%)
Other	8 (4.06%)	2 (1.18%)

SPALS: Single-port access laparoscopic surgery, SSRS: single-site robotic surgery.

**Table 2:** Baseline characteristics.

	SPALS (n = 197)	SSRS (n = 170)	*p* Value
Age (years)	44.5 ± 12.3	39.8 ± 9.54	0.001
BMI (kg/m^2^)	23.4 ± 3.64	22.9 ± 3.76	0.191
Parity	1.49 ± 1.09	1.11 ± 1.03	0.08
Diabetes	3 (1.5%)	6 (3.5%)	0.313
Previous surgery (n)	88 (44.7%)	63 (37.1%)	0.167
Pelvic adhesion (n)			
No	127 (64.7%)	103 (60.6%)	0.004
Mild	36 (18.3%)	15 (8.8%)	
Moderate	16 (8.1%)	25 (14.7%)	
Severe	18 (9.1%)	27 (15.9%)	

SPALS: single-port access laparoscopic surgery, SSRS: single-site robotic surgery, BMI: body mass index.

**Table 3:** Surgical outcomes.

	SPALS (n = 197)	SSRS (n = 170)	*p* Value
Total operation time (min)	57.1 ± 27.28	118.1 ± 65.95	0.001
Hb change after surgery	1.36 ± 1.05	1.5 ± 1.00	0.211
Postoperative pain score (NRS)	2.8 ± 1.09	2.92 ± 1.10	0.114
Surgery-to-gas passing time (hours)	30.4 ± 13.53	39.4 ± 15.367	0.001
Intraoperative complications	4 (2%)	3 (1.8%)	0.853
Wound dehiscence	2 (1.02%)	5 (2.94%)	0.336
Wound hernia	1 (0.51%)	2 (1.18%)	0.898
Hospital stays	4.02 ± 0.82	4.26 ± 1.02	0.012
Readmission rates within 1 month	3 (1.52%)	0 (0%)	0.301
Medical cost	$1170 ± 492	$7221 ± 684	<0.001
Fertility-preserving surgery	37 (18.78%)	120 (70.58%)	<0.001

SPALS, single-port access laparoscopic surgery; SSRS, single-site robotic surgery; NRS, numeric rating scale.

**Table 4:** Univariate and multivariate regression analyses of operation time and surgery-to-gas passing time.

	Univariate			Multivariate		
Variable	Regression Coefficient	SE	*p* Value	Regression Coefficient	SE	*p* Value
Operation time						
BMI	1.111	0.817	0.175			
Diabetes	33.786	19.435	0.083			
Previous surgery	−8.515	6.118	0.165			
Age	−0.129	0.261	0.621			
Parity	−5.791	2.785	0.038	−0.857	2.421	0.724
Adhesion	3.953	2.800	0.159			
Operation	60.985	5.143	0.000	60.660	5.231	0.001
Surgery-to-gas passing time						
BMI	−0.001	0.225	0.996			
Diabetes	15.779	5.401	0.004	12.322	5.209	0.019
Previous surgery	0.427	1.734	0.806			
Age	−0.330	0.077	0.665			
Parity	0.732	0.788	0.354			
adhesions	2.126	0.778	0.007	1.437	0.753	0.050
Operation	9.031	1.619	0.000	8.312	1.616	0.001

SE, standard error; BMI, body mass index.

## Data Availability

Data are available from the authors upon reasonable request.
